# Estimating the prevalence and intensity of *Schistosoma mansoni* infection among rural communities in Western Tanzania: The influence of sampling strategy and statistical approach

**DOI:** 10.1371/journal.pntd.0005937

**Published:** 2017-09-21

**Authors:** Jared S. Bakuza, Matthew J. Denwood, Gamba Nkwengulila, Barbara K. Mable

**Affiliations:** 1 Institute of Biodiversity Animal Health and Comparative Medicine, College of Medical, Veterinary and Life Sciences, University of Glasgow, Glasgow, United Kingdom; 2 Department of Biological Sciences, Faculty of Science, Dar es Salaam University, College of Education, Dar es Salaam, Tanzania; 3 School of Veterinary Medicine, College of Medical, Veterinary and Life Sciences, University of Glasgow, Glasgow, United Kingdom; 4 Department of Veterinary and Animal Sciences, Faculty of Health and Medical Sciences, University of Copenhagen, Copenhagen, Denmark; 5 Department of Zoology and Wildlife Conservation, College of Natural and Applied Sciences, University of Dar es Salaam, Dar es Salaam, Tanzania; Durham University, UNITED STATES

## Abstract

**Background:**

*Schistosoma mansoni* is a parasite of major public health importance in developing countries, where it causes a neglected tropical disease known as intestinal schistosomiasis. However, the distribution of the parasite within many endemic regions is currently unknown, which hinders effective control. The purpose of this study was to characterize the prevalence and intensity of infection of *S*. *mansoni* in a remote area of western Tanzania.

**Methodology/Principal findings:**

Stool samples were collected from 192 children and 147 adults residing in Gombe National Park and four nearby villages. Children were actively sampled in local schools, and adults were sampled passively by voluntary presentation at the local health clinics. The two datasets were therefore analysed separately. Faecal worm egg count (FWEC) data were analysed using negative binomial and zero-inflated negative binomial (ZINB) models with explanatory variables of site, sex, and age. The ZINB models indicated that a substantial proportion of the observed zero FWEC reflected a failure to detect eggs in truly infected individuals, meaning that the estimated true prevalence was much higher than the apparent prevalence as calculated based on the simple proportion of non-zero FWEC. For the passively sampled data from adults, the data were consistent with close to 100% true prevalence of infection. Both the prevalence and intensity of infection differed significantly between sites, but there were no significant associations with sex or age.

**Conclusions/Significance:**

Overall, our data suggest a more widespread distribution of *S*. *mansoni* in this part of Tanzania than was previously thought. The apparent prevalence estimates substantially under-estimated the true prevalence as determined by the ZINB models, and the two types of sampling strategies also resulted in differing conclusions regarding prevalence of infection. We therefore recommend that future surveillance programmes designed to assess risk factors should use active sampling whenever possible, in order to avoid the self-selection bias associated with passive sampling.

## Author summary

Schistosomiasis constitutes a major public health problem in Tanzania, with up to 80% prevalence of infection in some areas. Infection with the disease causes abdominal pain, diarrhoea, stunted growth and impaired cognitive abilities in children. Accurate information on the distribution of schistosomiasis within Tanzania is not available for most rural areas. To establish the prevalence of the intestinal form of the disease among communities residing along the shores of Lake Tanganyika, we quantified the presence of the causal agent (*Schistosoma mansoni*) by searching for their eggs in faecal samples. Children were actively sampled from schools but adult sampling relied on volunteers, so the data from the two age groups were analysed separately. The number of positive samples was high in both but significant differences in prevalence and intensity were found between the sampled villages only for the children. Statistical models accounting for false negative stool samples indicated that the apparent prevalence was a gross under-estimate of the true prevalence of infection. Our results emphasise the importance of considering the type of sampling when assessing risk factors associated with parasitic disease. The information obtained from this study will help to guide the optimal distribution of schistosomiasis control resources in the region, such as through targeted allocation of drugs and personnel to those areas with higher estimated prevalence.

## Introduction

Schistosomiasis, which is caused by trematode parasites in the genus *Schistosoma*, is a typically chronic disease that can result in debilitation and severe pathology in infected patients [1]. Infection with the parasite can also be asymptomatic and can remain undetected for a long period of time [2, 3], particularly when presenting intestinal schistosomiasis. This can lead to complacency and tolerance of the disease by both patients and the community. Thus, the disease does not receive as much treatment or financial support as malaria, tuberculosis and HIV/AIDS [4]. This has led the World Health Organisation to categorise schistosomiasis as a Neglected Tropical Disease [5].

Both *S*. *mansoni* (which typically causes intestinal schistosomiasis) and *S*. *haematobium* (now termed urogenital schistosomiasis) are endemic throughout Tanzania, with a prevalence of up to 80% in some areas [6–8]. The disease constitutes a major public health problem [9], but the control efforts have been limited by a lack of reliable data on the distribution and prevalence of the parasite across different parts of the country. Recent estimates indicate that *S*. *haematobium* infection is distributed mainly along the coast of the Indian Ocean and in inland villages around Lake Victoria [10], while *S*. *mansoni* infections have been reported in most parts of the country except the eastern coastal areas and Zanzibar and Pemba islands [3, 10, 11]. However, reliable data on schistosomiasis infection in Tanzania are mostly limited to the northeast and Lake Victoria areas, which have been more extensively studied due to their accessibility and more advanced infrastructure compared to other parts of the country [10]. Similar information is currently lacking for the southern and western areas, including the Kigoma District. In these areas, information on schistosomiasis comes mostly from hospital records, but as a consequence of poor recording and non-random sampling [12], this information gives biased estimates of population health attributes such as prevalence and infection intensity [13].

Furthermore, a general limitation of assessing the impact of parasitic infections is the typically highly aggregated nature of the data, and the statistical models that these characteristics demand. Schistosomes tend to show over-dispersion in abundance, with some host individuals having very high observed faecal egg or adult worm counts and others having few or zero counts, which is typical of many parasite species [14]. For some such data, a zero-inflated negative binomial (ZINB) distribution has also been used [15, 16], with a proportion of the ‘zero’ count observations described by a latent class of individuals that are not infected with the parasite, and the remainder of the ‘zero’ count observations obtained from the negative binomial distribution describing the infected individuals. Standard analytical approaches have often assumed a Poisson or negative binomial distribution for count data but have tended to separately estimate the prevalence based on the proportion of observed counts above zero. This under-estimates the true prevalence because of the imperfect sensitivity of egg detection in infected individuals [17, 18]. It may therefore be preferable to use a ZINB distribution to allow simultaneous consideration of both the skewed underlying distribution, including zero counts from infected individuals, and the proportion of truly uninfected individuals [19]. This allows a less biased estimation of prevalence because it does not assume that all ‘zero’ count samples represent uninfected individuals; rather, it allows for some parasite infections to be present but not detected. This is reflected in the zeros expected under a negative binomial distribution: in a theoretical population where all individuals are infected with equal numbers of parasites and a standard Kato-Katz test is applied, a distribution of counts would arise due to the non-random distribution of parasite eggs in faeces. This distribution may well contain zeros arising from the imperfect sensitivity of the diagnostic method, but these would not reflect uninfected individuals: they are in fact ‘false negative’ counts. Conversely, a ZINB model reflects two underlying processes: an infected/uninfected status for each individual whereby each uninfected individual must have a count of zero (the ‘extra’ zeros as estimated by the zero-inflation part of the model), and a distribution of observed counts from the infected individuals, which may take any positive discrete value including zero (the negative binomial part of the model). Therefore, each of the zero observations may actually be derived from either uninfected (zero-inflated) or infected (negative binomial) individuals. Extending this principle, such models can use the model’s zero-inflation and negative binomial terms to separate the factors affecting the presence/absence of infection in the host (or more correctly, infection with adult female parasites) from the factors affecting the distribution of egg shedding intensity between infected hosts. This is done by estimating the effects of a set of potential risk factors for the degree of zero-inflation using a logistic regression model (binomial response with logit link), and separately estimating the effects of a set of potential risk factors describing the intensity of observed counts from infected individuals using a negative binomial regression (typically with a log link). Either the same set of risk factors can be used for these two parts of the model, or different sets of risk factors can be used where there is an *a priori* justification for excluding a risk factor used in either the zero-inflation or negative binomial term from the other term.

Another factor to consider in parasite control measures is whether there are other reservoirs of infection that might maintain the disease even if all humans were treated [20]. For schistosomiasis, there is some evidence that non-human primates (mostly baboons and vervet monkeys) can harbour the same species of schistosomes as humans [21–24]. The prevalence of schistosomes in the wild animals is not clearly known, but neither is the prevalence in humans in major areas of contact such as at Gombe National Park in western Tanzania [22, 24]. Given the economic importance of primate ecotourism in Tanzania [25], an important knowledge gap to address is the potential schistosomiasis risk humans might pose to animals and the potential risk posed to tourists. The park was made famous by Jane Goodall for its chimpanzees and is now a popular tourist destination [26]. Although very little is known about schistosome prevalence or intensity in humans in this area [10, 22, 27], in an accompanying study we confirmed the presence of *S*. *mansoni* in the main vector of disease in the region, *Biomphalaria pfeifferi* [24]. The purpose of this study was to establish the distribution and prevalence of *S*. *mansoni* in humans residing within Gombe National Park and its surrounding villages, using appropriate sampling and analytical approaches.

## Methods

### Ethics statement

Ethical Clearance for this study (No. NIMR/HQ/R.8a/Vol.IX/892) was issued by the Tanzania’s National Institute for Medical Research (NIMR). The permission to survey schistosomiasis in villages and schools in the study area was obtained from the Tanzania Commission for Science and Technology (COSTECH) through the Executive Director of Kigoma District (Ref. No. KDC/G1/6/70). Before collecting stool samples full consent was obtained from each participant. A meeting was held with prospective participants who were informed in Kiswahili (the official language used in the study area) about the goals of the study, their voluntary participation and its implications. Those willing to participate in the study were asked to give consent through writing (signature) and oral for those who could not read or write. Parents, guardians and teachers gave consent on behalf of the children involved in the study. The WHO recommended dose of anthelmintics was given to all consenting individuals infected with schistosomes and other helminths.

### Study area

**Fig 1:**
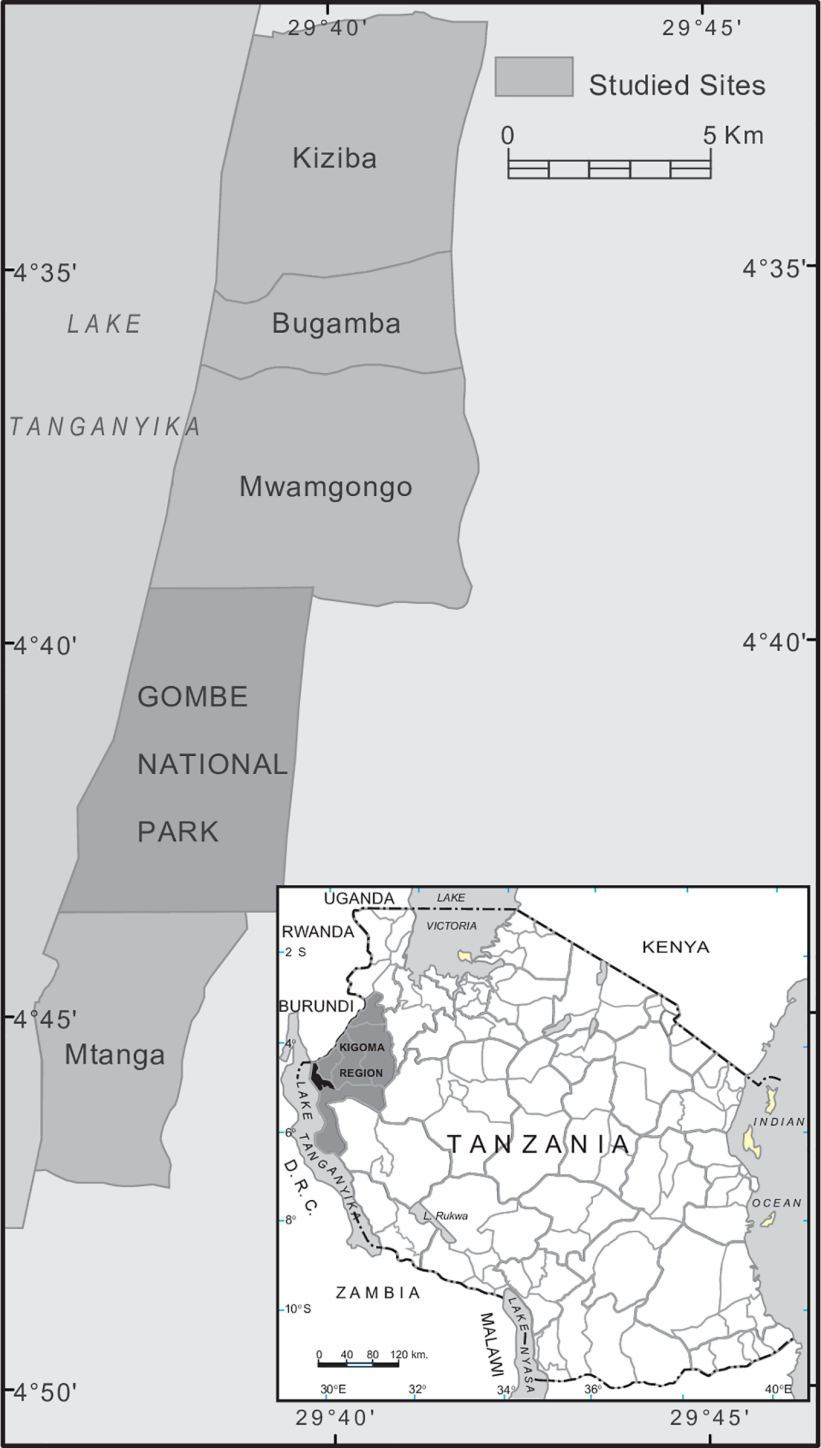
Map of the study area, showing the arrangement of villages sampled in relation to Gombe National Park. The inset shows the location of the study area within Tanzania.

This study was conducted in Gombe National Park and the neighbouring villages to the north (Kiziba, Bugamba, Mwamgongo) and south (Mtanga), along the eastern shores of Lake Tanganyika in the Kigoma District (Fig 1). Gombe National Park (4◦53′ S, 29◦38′ E) is a narrow strip of rugged terrain and hills along the shores of Lake Tanganyika [24, 28]. The top of the hills forms the park’s eastern boundary while the lakeshore forms its western boundary. The park is directly bordered by Mtanga and Mwamgongo villages, each of which are inhabited by approximately 5000 people, with most of them engaged in fishing activities in Lake Tanganyika [29]. The more northern villages (Bugamba and Kiziba) each harbour about 10,000 residents, the majority of whom are farmers [30]. Each of the studied villages has at least one stream that runs through it, where *B*. *pfeifferi* snails known to transmit schistosomes in the area have been identified [24, 30].

### Sampling strategies

Two separate sampling strategies were employed for this study. The first dataset was obtained by active sampling of a target of 60 school children selected from "standard three" classes at a single school per village. This class was chosen because the pupils represent the median age of primary school children in Tanzania (9–12 years), and also so that the results obtained would be comparable with other studies that have been used to inform control programmes [31]. In schools where standard three class had fewer than 60 children, additional pupils were recruited from standard four class. There was no primary school in Gombe National Park at the time of study, so this site was not included in the statistical modelling for the active sampling dataset.

The second dataset was obtained by passive sampling of adult individuals presenting voluntarily at the village clinics, which is therefore potentially subject to self-selection bias. Village sub-divisions were used as selection units for adults, with a cut-off point of at most ten individuals from each sub-division to ensure equal village representation. At Gombe, sampling was conducted in the main residence areas of Kalande near the park’s southern boundary, Kasekela in the centre of the park and Mitumba near its northern boundary. At each site, any dependent children accompanying adults to the clinic (“accompanying children”) were also sampled. These were not included in the statistical modelling due to small sample sizes and potentially different self-selection bias, so egg counts are reported for qualitative comparison only. This included all nine children above 12 months of age who were resident in Gombe at the time of sampling, because there were no schools to be sampled.

Sampling was conducted in 2010 during the wet season (January to April). For adults, consenting participants were registered, weighed and their age and sex recorded. For school children, age was obtained from the school register while for accompanying children, their medical clinic cards were used to estimate their age.

Sampling kits and instructions on the collection protocol were distributed in the morning of the first sampling day and the samples collected back in the morning of the following day. Each sampling kit was comprised of a wooden spatula for picking up a stool sample, a pre-labeled plastic vial (120 ml) for depositing the stools and a locally made polythene plastic bag for keeping the samples. Infection status was based on a single sample from each individual and a single Kato-Katz slide per sample. The WHO recommends that samples are obtained over three days and multiple slides counted per sample [31] to avoid underestimating prevalence and over-estimating intensity of infections [32–34], so our approach is conservative as a first assessment of whether schistosomiasis is present at a substantial level in the region. This approach has been used by previous epidemiological studies [35] and we followed recommendations to scan entire Kato-Katz slides rather than extrapolating from sampling a subset of slides, to increase accuracy [36]. We have also employed appropriate distributions in our statistical analyses to fully account for the imperfect sensitivity of egg detection in faecal samples.

### Laboratory procedures

Stools were examined for *S*. *mansoni* and other helminths using the Kato-Katz kit, following the manufacturer’s guidelines (Bio-Manguinhos, Rio de Janeiro, Brazil) and descriptions in the literature [31, 36, 37]. The faecal material was first pressed through a mesh screen filter to remove large particles. The filtrate was then transferred onto a microscope slide through a template hole that holds 41.7 mg of faecal material, which is a recommended standard [37, 38]. The template was then removed and a hydrophilic cellophane paper previously soaked in glycerol-malachite green solution placed on the faecal material. A second slide was placed onto the cellophane strip and pressed to spread out the sample for easy observation of parasite eggs. The bottom slide was left to clear for 30–60 minutes. The slide was then placed under a compound microscope and the entire preparation examined using a 10x objective and the parasite identified using 40 x objectives. All schistosomes and other helminth eggs observed on each slide were identified and counted based on standard guidelines [24, 37, 39]. The eggs of *S*. *mansoni* were easily identified based on their distinguishing lateral spines, while other helminth eggs were identified based on their morphology, size and appearance of eggs/larvae. The observed number of schistosome eggs observed per Kato Katz slide (holding 41.7 mg of faeces) was recorded as raw faecal worm egg counts (FWEC), and also converted to the standardised eggs per gram (EPG), as typically used in the literature by simply multiplying the FWEC by 24. EPG is used as a proxy for estimating the intensity of parasitic infections since it is related to total worm count but can be readily estimated from live patients [31, 40–44]. We refer to the proportion of samples with EPG greater than zero as the “apparent prevalence” to distinguish it from the prevalence as estimated by the statistical models, which take into account uncertainty about which zero counts reflect true absence of eggs and which represent lack of detection of eggs. The observed FWEC were used for the statistical models instead of the EPG in order to fulfil the requirement that the response for the statistical model is distributed according to a count [45], and where appropriate, model estimates were transformed to the equivalent scale as EPG by multiplying by the same constant of 24.

### Quantitative analysis

Because of the fundamental difference in the sampling procedures between the actively sampled (school children) and passively sampled (self-selected adults) data, the two datasets were analysed separately, but using the same procedure. A negative binomial model was first fit to the data, using a stepwise algorithm to select the best-fitting model from the four possible explanatory variables of site, sex, linear effect of age, and quadratic effect of age. Sex and site were fitted as categorical variables with 'Male' as the reference category for sex, and the site with the highest number of observations for each dataset chosen as the reference category (Kiziba for the actively sampled dataset and Mwamgongo for the passively sampled dataset). Once the best fitting negative binomial (NB) model had been found, two zero-inflated generalisations of this selected negative binomial model were tested: first using only an intercept term in the zero-inflation part of the model (ZINB1), and secondly using the same predictors in the zero-inflation part of the model as were used in the negative binomial part of the model (ZINB2). Both ZINB models allow the underlying distribution of FWEC to be conceptually split into two groups: those individuals belonging to the egg shedding group, and those individuals belonging to the ‘zero’ group. This assumes that all samples containing one or more observed eggs originated from an individual in the infected group, whereas samples containing no eggs could have originated from either an infected individual, from whom a positive sample may have been obtained on another day, or from an individual in the ‘zero’ group, from whom it is not theoretically possible to obtain a sample containing eggs. Using this distribution effectively allows separate generalized linear models to be fit simultaneously to the intensity of egg shedding in infected individuals, using a negative binomial distribution with log link, and to the prevalence of egg shedding between individuals, using a binomial distribution with logit link. The ZINB1 model allows for a set of extra zeros that do not belong to the distribution of 'infected' individuals, with an equal probability of each individual being in this extra zero set independent of the predictors in the model. The ZINB2 model allows the probability of each individual being in the extra zero set to depend on the explanatory variables also used for the NB part of the model. Both ZINB1 and ZINB2 models collapse to the NB model in the special case that the extra-zero component is estimated to be negligible (i.e. prevalence is estimated to be close to 100%).

All statistical analyses were performed in R Version 3.2.2 [46]. The NB models were fit using the glm.nb function of the MASS package [47], and the step-wise selection algorithm was based on the Akaike Information Criterion (AIC) [48]. Models including zero-inflation terms were fit using the zeroinfl function in the pscl package [49]. Assessment of model fit for zero-inflated models is not valid using AIC, so two alternative approaches were used based on: (a) the Vuong statistic [49]; and (b) a distribution of 1000 likelihood ratio test statistics obtained from data generated under the NB model [50]. The ZINB models were only considered preferable to the NB model in the case that both fit statistics indicated that this was the case.

## Results

### Qualitative descriptions of the data

A total of 198 and 149 FWEC observations were made for the actively and passively sampled datasets respectively, of which 105 and 54 were counts of greater than zero, corresponding to apparent prevalence of 53% and 36% (Table 1). For accompanying children, 35 individuals were sampled, of which 15 had egg counts greater than zero, corresponding to an apparent prevalence of 43%. The number of participants and ratio of adults to children varied substantially between sites, with Mwamgongo showing the highest number of people who participated in the study and the highest apparent prevalence compared to the other villages (Table 1 and S1 Table).

**Table 1:** Summary of apparent prevalence of schistosome eggs in participants sampled from Gombe National Park and its neighbouring villages: actively sampled (school children), passively sampled (self-selected adults), and accompanying children (non-school children that were sampled along with the passively sampled adults).

	Kiziba^a^	Bugamba	Mwamgongo	Gombe	Mtanga
Passively sampled	5/12 (8)^b^	8/17 (7)	20/47 (18)	11/37 (13)	10/36 (21)
Actively sampled	19/56 (20)	38/53 (28)	42/49 (27)	--^c^	6/40 (24)
Accompanying children	4/6 (1)	1/3 (1)	9/9 (7)	1/9 (5)	0/8 (2)

^a^ Sites are ordered from north to south along the shores of Lake Tanganyika

^b^ The numerator indicates the number of egg counts greater than zero, while the denominator is the number of individuals sampled per site for each dataset; the number of females sampled is indicated in parentheses

^c^ There was no active sampling in Gombe because it does not contain a school.

For accompanying children, although sample sizes were small, Mwamgongo also showed higher apparent prevalence than the other sites (Table 1).

**Fig 2:**
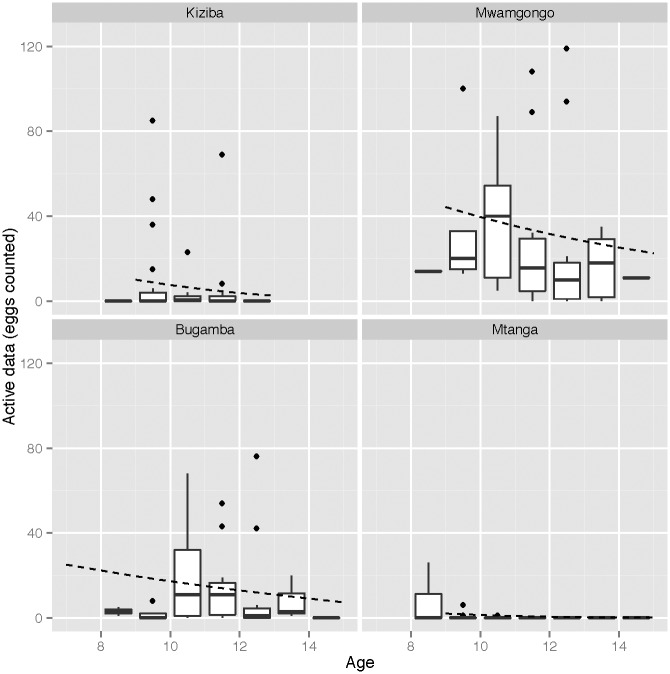
Box and whisker plots of the observed faecal egg counts within each age category for each site for the actively sampled data. The overlaid dashed line shows the relationship between age and site for the truly infected individuals, as estimated by the ZINB2 model.

**Fig 3:**
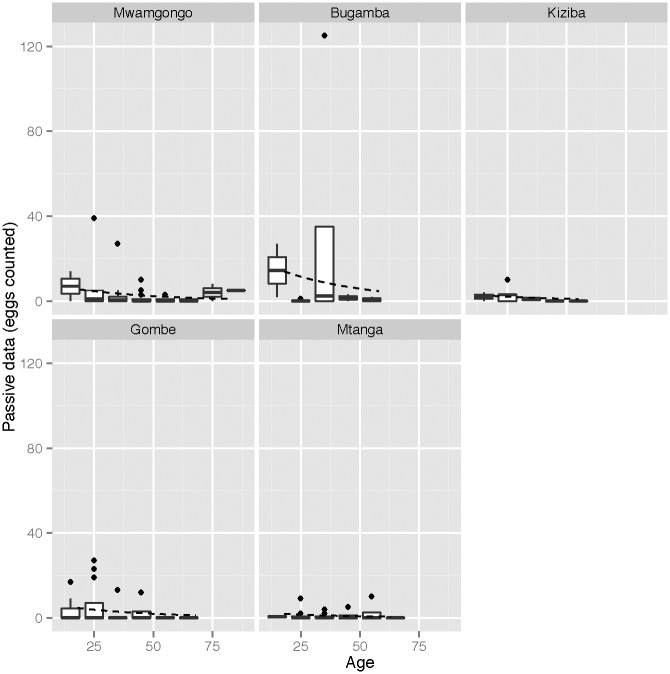
Box and whisker plots of the observed faecal egg counts within each age category (grouped into intervals of 10 years) for each site for the passively sampled data. The overlaid dashed line shows the relationship between age and site as estimated by the NB model.

Qualitatively, there was substantial variation in observed FWEC between sites, and variation between age groups for some sites (S1 Table, Figs 2 and 3). There were also differences between male and female adults at some sites. For example, at Bugamba, adult females showed lower apparent prevalence and lower FWEC than adult males or children. This was not the case at Mwamgongo, where females with non-zero counts showed higher loads than males. Overall, the qualitative data suggest high variance among individuals and sites.

### Quantitative analyses

#### Actively sampled dataset

The stepwise selection procedure indicated that the most parsimonious NB model included effects of site and a linear effect of age, but not the effect of sex or a quadratic effect of age. The ZINB1 model was not preferred based on either the Vuong statistic (p = 1) or the bootstrapped LRT (p = 0.85). The ZINB2 model was preferred to the NB model based on both the Vuong statistic (p = 0.001) and the bootstrapped LRT (p<0.001). The final model chosen was therefore the ZINB2 model, with effects of site and age in both the zero-inflation (ZI) and intensity (NB) terms within the model (Table 2).

**Table 2:** Fitted estimates and p-values for the relevant effects estimated from the final ZINB2 model for the actively sampled data.

	Intensity Term (negative binomial)		Zero-inflation Term (true prevalence)	
	Estimate	p-value	Estimate	p-value
Kiziba	0	--	0	--
Bugamba	0.24 (-0.57–1.0)	0.565	-2.85 (-4.97–-0.72)	0.009
Mwamgongo	1.02 (0.17–1.9)	0.019	-12.20 (-231.58–207.18)	0.913
Mtanga	-0.98 (-2.32–0.37)	0.156	1.19 (-0.082–2.45)	0.067
Age (years)	-0.11 (-0.35–0.13)	0.361	0.41 (-0.14–0.96)	0.142

The log mean number of counted eggs at the reference site (Kiziba) was estimated at 2.49 (corresponding to 12 counted eggs or 264 EPG), with an inverse over-dispersion parameter (*k*) of 0.48. The effect of age was not found to be significant in either the prevalence or intensity terms. There was a significant difference in the mean count and prevalence between some sites, with a significantly higher prevalence at Bugamba compared to Kiziba (p = 0.009), and a significantly higher mean count in the infected individuals in Mwamgongo compared to Kiziba (p = 0.019).

#### Passively sampled dataset

The stepwise selection procedure indicated that the most parsimonious NB model included effects of site and a linear effect of age, but not sex or a quadratic effect of age. Neither the ZINB1 model nor ZINB2 model were preferred based on either the Vuong statistic (p = 1 and p = 0.14, respectively) or the bootstrapped LRT (p = 0.79 and p = 0.82, respectively). The final model chosen was therefore the NB model (Table 3).

**Table 3:** Fitted estimates and p-values for the relevant effects estimated from the final NB model for the passively sampled data. Note that there are no zero-inflation terms for the NB model because any observed zeros are assumed to reflect a failure to detect eggs in truly infected individuals.

	Intensity Term (negative binomial)	
	Estimate	p-value
Kiziba	-0.80 (-2.45–1.30)	0.372
Bugamba	0.91 (-0.48–2.54)	0.211
Mwamgongo	0	--
Gombe	-0.18 (-1.41–1.04)	0.757
Mtanga	-1.09 (-2.25–0.10)	0.069
Age (years)	-0.03 (-0.06–0.003)	0.102

The log mean number of counted eggs was estimated at 1.12 (corresponding to 3 counted eggs or 74 EPG), with an inverse over-dispersion parameter (*k*) of 0.17. There was no significant effect of age (p = 0.102) or site (overall p = 0.07) for the adult data, although both were included in the most parsimonious model based on model fit, and the estimated trend towards a decrease in intensity of egg shedding with increasing age is consistent with that estimated from the child data.

#### Estimated prevalence

The ZINB2 model was used to extract unbiased estimates for the site-specific prevalence. Note that the most parsimonious model for the passively sampled data was the NB model, which does not include a zero-inflation term and therefore implies that the prevalence is close to 100% in adults for all sites. However, for the purposes of comparison with the actively sampled data, and the apparent prevalence obtained from the raw data, we also used the ZINB2 model to estimate site-specific prevalence for the passively sampled data (Table 4).

**Table 4:** The apparent prevalence based on the raw data and estimated prevalence based on the ZINB2 model for the actively sampled (school children) and passively sampled (self-selected adults) data.

	Apparent Prevalence (%)		Estimated Prevalence (%)^a^ (95% CI)	
	Active	Passive	Active	Passive
Kiziba	35	45	59 (49–71)	99 (45–100)
Bugamba	73	47	98 (89–100)	89 (54–99)
Mwamgongo	89	43	99 (89–100)	99 (43–100)
Gombe	--	30	--	66 (44–88)
Mtanga	15	29	28 (19–47)	82 (38–99)

^a^ Estimates from the model are for an individual with age 11.5 (actively sampled data) and 40 (passively sampled data), corresponding to the average observed age for that dataset.

The estimated true prevalences were far higher than the biased estimates in every case, although the 95% confidence intervals are generally wide. There was also a corresponding change in the estimated mean intensity of infection for infected individuals based on the zero-inflated models compared to the simple summaries of the observed data (Table 5).

**Table 5:** The apparent intensity based on the raw data and estimated intensity of shedding for infected individuals based on the ZINB2 model for the actively sampled (school children) and passively sampled (self-selected adults) data, in eggs per gram (EPG).

	Apparent Intensity (EPG)		Estimated Intensity (EPG)^a^ (95% CI)	
	Active	Passive	Active	Passive
Kiziba	138.8	48.0	291 (150–564)	35 (9–138)
Bugamba	321.9	234.4	368 (232–584)	236 (65–854)
Mwamgongo	741.4	68.4	802 (506–1271)	72 (38–137)
Gombe	--	84.3	--	113 (37–344)
Mtanga	30.0	24.7	110 (31–391)	32 (11–97)

^a^ Estimates from the model are for an individual with age 11.5 (actively sampled data) and 40 (passively sampled data), corresponding to the average observed age for that dataset.

## Discussion

### Qualitative assessment of schistosome loads in the Gombe area

This study represents the first large-scale attempt to quantify the prevalence and intensity of schistosome infection in the Gombe ecosystem in western Tanzania. Overall, the results suggest that schistosomiasis could pose a substantial threat to human health in this under-sampled region. Despite the previous impression that the disease is rare in this region [10], Mwamgongo village, for instance, showed an apparent prevalence of more than 89% in children and an estimated true prevalence of approximately 100% for both adults and children, which is far higher than the estimated national average of 51.5% [51]. Although observed FWEC suggested extensive variation among villages, estimation of true prevalence of infections using zero-inflated models suggested that all of the sampled villages had a high proportion of individuals with schistosome infections, although with a low mean egg shedding rate in passively sampled adults relative to actively sampled children.

Encouragingly, the data suggest that prevalence of schistosomiasis is lower for the residents of Gombe National Park, although the estimated true prevalence still indicates that at least two thirds of adults are infected. This is despite the finding that the closest villages to the north (Mwamgongo and Bugamba) showed high prevalence and intensity of infection, both in terms of observed FWEC and parameters estimated from the models (Table 1 and S1 Table). Within the park, low infection levels could be due to the transient nature of residents (who might originate from other regions where schistosomiasis prevalence is lower or where treatment is more readily available) but there does not seem to be spill-over from the neighbouring villages. This also suggests that interactions between humans and potential primate reservoirs (the baboons) are not posing a major risk factor either to humans or to the wild animals in these regions. However, active sampling of school children was not possible in Gombe so these conclusions are based only on the passively sampled dataset. So, a difference in the magnitude of self-selection bias within Gombe relative to the other sites cannot be ruled out.

The apparent discordance in prevalence and intensity between the actively and passively sampled datasets within the same sites is also illustrative of the potential disadvantages of over-reliance on passively sampled data. Although school surveys using stool samples collected over three consecutive days is the WHO recommended protocol for prevalence and intensity of infection mapping [31], prevalence estimated for these remote areas tend to rely on passively sampled data from hospital records. The actively sampled data can be assumed to be a representative and random sample of the target population of school children within each site, which therefore gives an unbiased estimate of the prevalence and intensity of infection within this population. In contrast, the passively sampled data is subject to self-selection bias of unknown and inestimable magnitude, which is a problem that is well known within the field of surveillance [52]. Despite this, passively sampled data is frequently used because it is typically cheaper and easier to collect, particularly when the dataset has already been collected for other purposes [53]. Although the magnitude of the bias is variable, passive surveillance systems typically under-estimate prevalence of infections [54, 55]. In contrast, we found higher estimates of prevalence in the passively sampled data relative to the actively sampled child data, which could be explained by individuals that suspect themselves to be infected being more likely to present for sampling and treatment. It is also possible that the actively sampled data may itself be subject to some bias; for example, due to school absenteeism or clinically sick children not attending school. Since the target populations for active and passively sampled data are not identical (school children vs. the adult population), we cannot directly assess the consequences of relying on passive sampling alone for robust assessment of prevalence. It is also not possible to directly compare the relative prevalence in the two age groups.

### Insights from statistical modelling

Application of models that can separately estimate variables explaining variation in prevalence and intensity of parasite loads emphasised two important limitations of traditional approaches: 1) assessing prevalence based on the proportion of non-zero FWEC; and 2) confounding differences due to age with sampling strategies (active sampling of all individuals vs self-selected volunteers). The estimated prevalence based on the ZINB2 model for the child data was higher than the apparent prevalence, because a proportion of the zero counts were due to a low counting sensitivity. Note that the converse is not possible (observed eggs are never assumed to be false positives), so the apparent prevalence is an estimate of the true prevalence that is necessarily biased downwards. The ZINB2 model provided a significantly better fit to the data than the NB model for the actively sampled data, and there is evidence to suggest that the true infection prevalence amongst children is less than 100% in some villages. There was no evidence that the zero-inflated models produced a better fit to the passively sampled data than the simpler NB model, which means that all of the observed zero egg counts in this data were consistent with a failure to detect eggs in truly infected individuals due to imperfect sensitivity of egg detection. This could be interpreted as the data being consistent with a true infection prevalence of 100%. However, it is impossible to exclude the possibility that a zero-inflated model would provide a better fit to a larger dataset, and when the ZINB2 model is used, there is some evidence that the prevalence is less than 90% in Gombe.

Since we did not have a large enough sample size of accompanying children they were excluded from the statistical analyses, but for a future study it would be worth exploring the consequences of including different sampling schemes when age is not a confounding factor. Age has often been suggested as a risk factor for parasite infections, in relation to development of immunity and behaviours that increase risk of infection [56–60]. If we had considered only the raw FWEC counts or combined the two datasets, our conclusions might also have been that children tended to show higher infection levels than adults. However, the ZINB modeling suggested that true prevalence in adults was actually higher than in children at some sites, which is likely due to self-selection bias in the passively sampled data.

The significantly better fit of the ZINB2 model compared to the NB model for the actively sampled individuals also suggests that a single over-dispersed Poisson distribution is not able to adequately explain all of the zero count observations. Zero-inflated distributions have been used in the fields of parasitology [16, 61], bovine mastitis [62], forest science [63], and medical epidemiology [64], although their use has also been criticized in modeling road traffic accident analysis data [65]. They are conceptually useful when zero observations can arise from either count data (such as a Poisson, negative binomial or other distribution) or from a truly zero individual [19], and may be of value in other similar applications. Correct identification of the degree of zero-inflation depends on the correct choice of distribution for the infected group, so that the number of ‘expected’ zeros is accurate. The negative binomial distribution is widely used, but other distributions such as the lognormal-Poisson may be more correct for some datasets [66] and may influence the estimates for zero-inflation [67]. Considerable care should therefore always be put into selection of the most biologically sensible distribution model for analysis of count data, and if appropriate some consideration towards the possibility of using a zero-inflated model should be made. In this case, there is a biologically plausible explanation for modeling a process where some individuals are uninfected, with a distribution of FWEC between the infected individuals, so the choice of ZINB model is justified.

A single Kato Katz slide was used to estimate the infection intensity, combined with rigorous statistical analysis to overcome the differences in sensitivity for prevalence and infection intensity of *S*. *mansoni* in epidemiological studies. A larger number of Kato Katz slides (as recommended by WHO guidelines [31]) would have decreased the relative size of the 95% confidence intervals by adding more information to the data. However, the coefficient estimates obtained should not be biased by the reduced sample size, except for the estimate of the over-dispersion parameter *k*, which partly reflects the variability between samples and would therefore be affected by the increased precision associated with more slides. In contrast, the bias in the apparent prevalence estimates would be expected to decrease as the number of slides was increased due to the increased probability of detecting eggs, and therefore decreased false negative rate. This reflects the difficulties in interpreting apparent prevalence, and emphasises the value of an unbiased estimate of the prevalence such as that given by the ZINB model.

### Risk factors associated with schistosome infection in the Gombe region

Based on the ZINB and negative binomial models respectively, there was no significant effect of sex on FWEC within either the actively or passively sampled datasets. Previous studies have also not found any effect of sex on parasitic infections and predicted that other factors may be determining the infection levels [68–70]. However, since schistosomiasis transmission is highly related to occupational activities and water contact, in areas where fishing or farming are mostly done by women, such as among the Mende people in the Sierra-Leone, higher prevalence of schistosomiasis has been reported among females than males [71]. It is therefore possible that both men and women in the Gombe ecosystem, their social and cultural duties notwithstanding, are equally exposed to schistosome transmission in the local streams. As there is an insufficient supply of running tap water in the studied villages, both women and men have to use the streams or lake, albeit for different purposes. While men often come in contact with the stream water for bathing and ablution before prayers for Muslims, women use the stream water mostly for performing domestic chores as well as bathing. Nevertheless, interpretation of results could be confounded by self-selection and a gender bias in the willingness of individuals to participate in voluntary studies for the passively sampled data. In our study, for example, there were 82 male and 67 female volunteers. However, such forms of bias should not be present in the actively sampled data.

We also found that, while there was a trend for decreasing raw FWEC in both children and adults (Figs 2 and 3), statistical models suggested a weak (although non-significant) effect of age in adults. Age-related differences in infection distributions have been found in other studies [56–60], where the prevalence and intensity of *S*. *mansoni* were found to rise slowly in children and then slowly decline in older individuals. It has been suggested that young people tend to be more susceptible to infection due to being in contact with water more often than adults [58, 72] and that the decrease in schistosome infection with age may be due to acquired immunity after repeated exposure [58, 72, 73]. Sampling of a broader range of age classes would be required to test these effects but with consistent sampling strategies across age groups. While difficult to implement, active sampling of adults would be required to fully test for age-specific differences in susceptibility.

The most pronounced differences in FWEC in our study appeared to be due to site. Although statistical modeling suggested that there were no dramatic differences among the villages, both raw egg counts and prevalence and intensity estimated from the ZINB models suggested differences between sites in children, with Bugamba and Mwamgongo standing out as the highest risk areas. In other communities living along the shores of Lake Victoria in northwest Tanzania, local variation in schistosome infections has been attributed to patchy distribution of snails [7, 74]. It is possible, therefore, that differences among the study villages in the present study could be due to differences in the resident snail populations. In a recent survey, Bakuza [24] did not find any infected snails in the streams sampled within the Gombe National Park boundaries and no snails at all were found in the stream running through Mtanga. This could help to explain the lower prevalence and intensity of infections at these sites. Further work is required to quantify whether differences in infection levels in humans are related to differences in snail densities in the various villages. Variation in schistosomiasis infection among villages could arise due to possible ecological risk factors, such as contact rates with infested water sources, human population density, socio-economic levels and differences in local snail ecology.

### Conclusions

Our findings offer some guidance on how to optimally distribute the limited resources for schistosomiasis control in areas along the shores of Lake Tanganyika, Tanzania and similar resource-poor settings in other endemic countries. Some differences between sites were found, which could be of relevance to designing future studies to improve understanding of the social and occupational risk factors for transmission of the disease. However, consistent with WHO guidelines, we recommend that surveillance should be conducted using active sampling wherever possible, to enable accurate estimation of prevalence and intensity. Moreover, we recommend that statistical models are applied based on the most appropriate distributions explaining the data. Compared to other studies, we found little influence of age or sex, which could reflect either differences between sampling procedures or real cultural or biological differences in the geographic region sampled for this study. Finally, qualitatively different prevalence estimates were obtained using the ZINB model compared to the observed prevalence based on the simple proportion of observed egg counts above zero, which demonstrates the potential for erroneous inference when ignoring the biasing effect of imperfect egg detection methods when estimating prevalence.

## Supporting information

S1 Table Overall raw mean egg counts of *S*. *mansoni* per village for adults and male and female school children across study sites in the Gombe area, Tanzania. Shown is the number of individuals sampled (N), the apparent prevalence based on egg count data (N>0), the mean number of eggs counted overall (Mean), the mean number of eggs only in individuals with counts greater than zero (Mean >0), the median, and the maximum egg count. Note that there is a strong apparent effect of site, with Bugamba and Mwamgongo showing higher egg counts than the other sites, particularly in children. Mtanga showed very low egg counts overall but some individuals still showed high intensity of infections. Among individuals showing greater than zero eggs counts, the highest parasite loads were in children from Mwamgongo, where there was also the largest difference between age classes in apparent prevalence.
(DOCX)

S2 Table Raw data used in the analyses presented. (XLSX)

S1 Checklist STROBE statement—Checklist of items that should be included in reports of *cross-sectional studies*. (DOC)
