# Supplementary material for: Estimating the prevalence and intensity of Schistosoma mansoni infection among rural communities in Western Tanzania: The influence of sampling strategy and statistical approach
Source: PLoS Negl Trop Dis. 2017 Sep 21;11(9):e0005937. doi: 10.1371/journal.pntd.0005937 (PMC5626504; doi:10.1371/journal.pntd.0005937)
Supplement: S1 Table — Shown is the number of individuals sampled (N), the apparent prevalence based on egg count data (N>0), the mean number of eggs counted overall (Mean), the mean number of eggs only in individuals with counts greater than zero (Mean >0), the median, and the maximum egg count. Note that there is a strong apparent effect of site, with Bugamba and Mwamgongo showing higher egg counts than the other sites, particularly in children. Mtanga showed very low egg counts overall but some individuals still showed high intensity of infections. Among individuals showing greater than zero eggs counts, the highest parasite loads were in children from Mwamgongo, where there was also the largest difference between age classes in apparent prevalence. (DOCX) [file pntd.0005937.s001.docx]

**Supplementary Table 1. Overall raw mean egg counts of *S*. *mansoni* per village for male and female school children and adults, across study sites in the Gombe area, Tanzania**. Shown is the number of individuals sampled (N), the apparent prevalence based on egg count data (N>0), the mean number of eggs counted overall (Mean), the mean number of eggs only in individuals with counts greater than zero (Mean >0), the median, and the maximum egg count. Note that there is a strong apparent effect of site, with Bugamba and Mwamgongo showing higher egg counts than the other sites, particularly in children. Mtanga showed very low egg counts overall but some individuals still showed high intensity of infections. Among individuals showing greater than zero eggs counts, the highest parasite loads were in children from Mwamgongo, where there was also the largest difference between age classes in apparent prevalence.

| **Site** | **Age_Class** | **Host_sex** | **N** | **N>0** | **Mean** | **Mean >0** | **Median** | **Maximum** |
| --- | --- | --- | --- | --- | --- | --- | --- | --- |
| Kiziba | adult | Female | 8 | 5 | 1.5 | 3.0 | 1 | 4 |
| Kiziba | adult | Male | 3 | 1 | 3.3 | 10.0 | 0 | 10 |
| Kiziba | child | Female | 20 | 8 | 5.3 | 13.3 | 0 | 48 |
| Kiziba | child | Male | 35 | 11 | 6.1 | 19.3 | 0 | 85 |
| Bugamba | adult | Female | 7 | 3 | 1.4 | 3.3 | 0 | 5 |
| Bugamba | adult | Male | 10 | 5 | 15.6 | 31.2 | 0 | 125 |
| Bugamba | child | Female | 28 | 24 | 17.7 | 20.7 | 11 | 68 |
| Bugamba | child | Male | 23 | 13 | 8.5 | 14.5 | 1 | 76 |
| Mwamgongo | adult | Female | 18 | 7 | 4.9 | 11.1 | 0 | 39 |
| Mwamgongo | adult | Male | 29 | 13 | 1.6 | 3.5 | 0 | 10 |
| Mwamgongo | child | Female | 27 | 23 | 23.8 | 27.9 | 14 | 94 |
| Mwamgongo | child | Male | 19 | 18 | 41.0 | 43.3 | 17 | 119 |
| Gombe | adult | Female | 13 | 4 | 4.4 | 14.3 | 0 | 19 |
| Gombe | adult | Male | 24 | 7 | 3.0 | 10.4 | 0 | 27 |
| Mtanga | adult | Female | 21 | 5 | 0.5 | 2.2 | 0 | 5 |
| Mtanga | adult | Male | 14 | 5 | 1.8 | 5.0 | 0 | 10 |
| Mtanga | child | Female | 24 | 5 | 2.0 | 9.8 | 0 | 26 |
| Mtanga | child | Male | 16 | 1 | 0.1 | 1.0 | 0 | 1 |
